# Concurrent proximal and distal fibular fracture with twisting ankle injury: a ‘Double Maisonneuve’ case report

**DOI:** 10.1016/j.tcr.2026.101319

**Published:** 2026-02-24

**Authors:** Stephen Christopher Murphy, Gareth Murray, Adeel Memon, Anas El-Sibaei

**Affiliations:** a Department of Trauma and Orthopaedics, Cork University Hospital, Whilton, Cork, T12 DC4A, Ireland

**Keywords:** Ankle fracture, Maisonneuve fracture, Preoperative CT, Surgical planning

## Abstract

**Background:**

Ankle fractures are a commonly encounter orthopaedic injury, with lateral malleolar fractures being the most common fracture pattern seen. Proximal fibular fracture with ankle twisting injuries, termed the Maisonneuve fracture, are much rarer, and are thought to account for 5% of surgical treated ankle fractures. Fracture of both proximal and distal fibular following twisting ankle injury is an extremely rare occurrence, with little reported cases in existing literature.

**Case presentation:**

We present one such case of a 45-year-old male who sustained both a proximal and distal fibular fracture following a twisting ankle injury with associated posterior malleolar fracture and anterolateral distal tibial tubercle (Tillaux-Chaput fracture). Both proximal and distal fractures were identified on presentation, and he underwent open reduction internal fixation with single cortical screw fixation of anterolateral distal tibial tubercle, lag screw to lateral malleolar and neutralization plate, and two tricortical syndesmotic screws. His post operative course was unremarkable, and he returned to work after nine weeks.

**Conclusion:**

Concurrent proximal and distal fibular fractures following ankle twisting injury is an extremely rare fracture pattern. This case highlights the necessity for thorough physical examination and radiological imaging for all ankle injuries while also highlighting the importance of preoperative computed tomography on surgical planning for ankle fractures.

## Background

Ankle fractures are common injuries, often requiring surgical fixation [1]. Owing to this fact, fractures involving the ankle have been studied copiously, and many physician lend their names to different fracture patterns, such as the Dupuytren, Chaput and Volkmann [2]. The Massioneuve fracture, described first in 1840 by Jules Germain François Maisonneuve, is another such eponyms ankle fracture associated with a proximal fibular fracture with rupture of the tibiofibular syndesmosis. This fracture pattern is thought to occur due to an external rotation force and is can be further classified by the Lauge Hansen classification as a Pronation External Rotation injury [3]. Such injuries are relatively infrequent, and account for about 5% of surgically treated ankle injuries [3].

An extremely unusual pattern of ankle fracture is a lateral malleolar fracture with cocurrent proximal fibular fracture. A case series involving 11 patients with such an injuries was recently described by Kasper et al. in 2022, however prior to this, only 4 cases were reported in the literature [4]. The authors coined this fracture pattern the ‘Double Maisonneuve’.

## Case report

We present the case of a 45-year-old male with an injury to his right ankle. He reported that he was assaulted by a security guard the previous evening where he was pushed over, twisting his right ankle in the process. Unfortunately, due to intoxication, he could not recollect exact mechanism or any further details. He awoke the following morning with pain and deformity in his ankle and was unable to weight bear. He hence presented to his local injury unit. Past medical history was significant for rheumatoid arthritis (RA) and left total hip arthroplasty (THA). He was an active smoker and worked as a machine operator on a building site.

On examination, he had a grossly swollen right ankle and diffuse tenderness, maximally over his lateral malleolus. He also reported pain and tenderness on the lateral aspect of his proximal fibular, just distal to the knee. There was no compromise to his soft tissues, and he was fully neurovascularly intact. He did not sustain any other injuries.

**Fig. 1:**
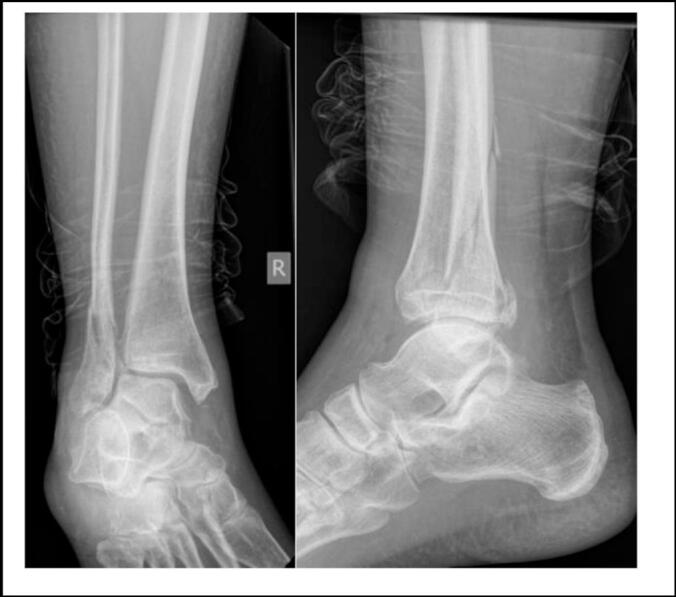
Initial AP and lateral ankle radiographs.

**Fig. 2:**
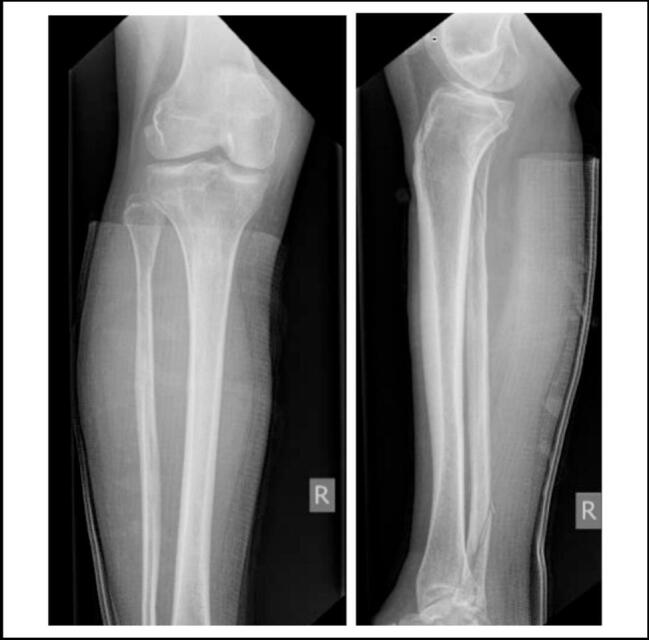
Initial tibial fibular radiographs.

Initial anteroposterior (AP), mortise and lateral radiographs demonstrated a distal spiral fibular fracture (Weber B), a small posterior malleolus fracture and a small avulsion fracture of the medial malleolus. Increased medial clear space and widening of the syndesmosis was also noted (Fig. 1). Further, full length tibial-fibular radiographs also revealed a spiral fracture of the proximal fibular around the fibular neck (Fig. 2).

**Fig. 3:**
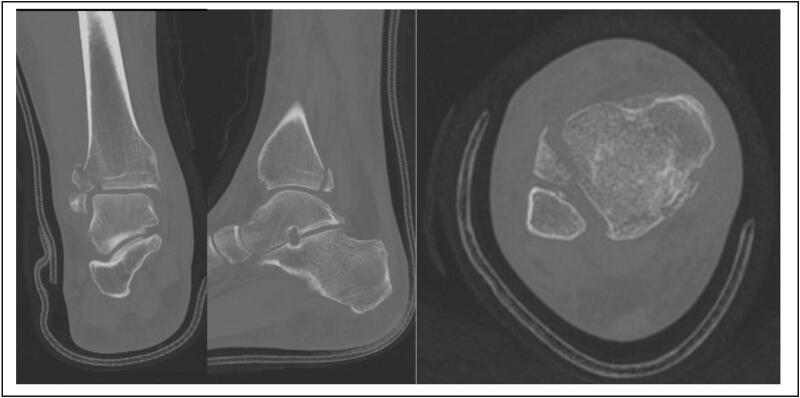
CT coronal, sagittal and axial views.

A computed tomography (CT) scan was subsequently arranged. This demonstrated the above findings with an additional finding of a moderately displaced fracture of the anterolateral distal tibial tubercle involving the tibial plafond (Tillaux-Chaput fracture) (Fig. 3).

After referral to our tertiary orthopaedic unit, he was placed into a below knee backslab and kept non-weight bearing (NWB). After consultation with our foot and ankle surgeon, open reduction internal fixation (ORIF) was arranged the in the following weeks as a day case procedure. He was discharged with analgesia and deep vein thrombosis (DVT) prophylaxis.

**Fig. 4:**
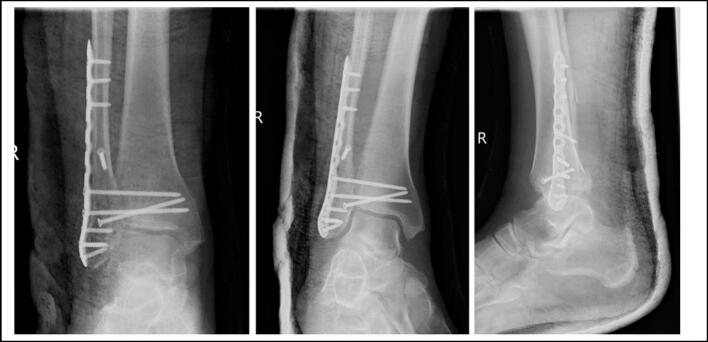
Initial post operative AP, mortice and lateral radiographs at 2 weeks post op.

Surgical fixation was done thirteen days following injury. Surgical fixation involved lag screw and neutralization plate for the distal fibula, a single percutaneous cortical screw for his anterolateral distal tibia fracture and two tricortical syndesmotic screws through the fibula neuralisation plate for syndesmotic injury (Fig. 4). Post operatively he was placed in a below knee backslab and maintained NWB.

He was seen two weeks post operatively. Radiographs and surgical site were satisfactory. He was placed in a walking boot and commenced on range of motion rehabilitation but kept strictly NWB. He was seen again at six weeks where he was progressed to full weight bearing (FWB) and discharged to physiotherapy.

At nine weeks post operatively, the patient report excellent function of his ankle and had returned to work.

A telephone consult was undertaken 1 year following surgery. The patient reported no issues with ankle function since his surgery and was very happy with the outcome.

## Discussion

Maisonneuve first described his eponym's fracture in 1840, and theorised it was secondary to an external rotation injury of the ankle [5]. Pankovich further described 5 stages of the development of the Maisonneuve fractures [6] as follows: 1) rupture of the anterior tibiofibular ligament, 2) rupture of the posterior tibial ligament, 3) rupture of the anteromedial insertion, 4) proximal fibular fracture and finally 5) rupture of the deltoid ligament [7], [8].

Due to the unique fracture pattern and clinical presentation seen with Maisonneuve fractures, its diagnosis may often be missed. In most cases, there is a fracture of the medial malleolus (or the deltoid ligament), posterior malleolus, or both. However, in rarer cases, a purely ligamentous injury can occur at the level of the ankle, leading to no fracture visible on ankle radiographs [6], [9]. Furthermore, patients will often not report any pain at the proximal fibula, possibly due to the distracting injury of the ankle or the relatively little weight forces transferred through the fibula, or both [5]. Due to the inherit instability of this fracture pattern, prompt diagnosis and appropriate management is essential for avoiding pain and preservation and restoration of function. This case highlights the importance of thorough clinical exam and radiological evaluation, especially examining for tenderness at the proximal fibular and full-length tibia fibular radiographs, to out rule a Maisonneuve or even Double Maisonneuve injury. Furthermore, due to the close anatomical relationship of the proximal fibula and the common peroneal nerve, high fibular fractures can cause injury to this nerve, typically presenting with diminished sensation over the dorsum of the foot and/or a foot drop [10]. This again highlights the importance of clinical exam and accurate diagnosis of these injuries.

When we exam biomechanics of the Massioneuve fracture, it is extremely unusual to fracture both proximal and distal aspects of the fibula. A literature review was undertaken to identify similar cases, of which 15 were found, 11 from a singled centre case series over 8 years [4], [7], [11], [12], [13] (Table 1).

**Table 1:** Literature review of previous proximal and distal fibular fractures.

	Paper, year	Age	Sex	MOI	Weber grade	MMF	PMF	TCF	Treatment	Delay in proximal fibular fracture Dx
1	Slawski, 1995 [7]	46	F	Slipped off curb	B	No	Yes	No	ORIF	No
2	Hensel, 2002 [12]	29	M	Sports injury	B	Yes	No	No	ORIF	No
3	Colenbrander, 2005 [11]	56	F	Slipped on wet step	C	No	No	No	ORIF	1 week
4	Wolfram, 2007 [13]	63	F	Fall down stairs	B	Yes	Yes	No	ORIF	3 days
5	Kasper, 2021 [4]	21	M	Fall from skateboard	C	No	Yes	No	ORIF	No
6	Kasper, 2021 [4]	44	M	Fall down stairs	C	No	Yes	No	ORIF	No
7	Kasper, 2021 [4]	56	F	Slip on ice	C	No	Yes	No	ORIF	No
8	Kasper, 2021 [4]	70	F	Fall from chair	C	Yes	Yes	No	EF + KW	No
9	Kasper, 2021 [4]	43	F	Slip in bathroom	C	Yes	Yes	No	ORIF	No
10	Kasper, 2021 [4]	76	F	Tripped	C	Yes	Yes	Yes	EF + ORIF	No
11	Kasper, 2021 [4]	53	F	Fall over dog	C	Yes	Yes	Yes	ORIF	No
12	Kasper, 2021 [4]	61	M	Slip on ice	C	No	Yes	Yes	ORIF	No
13	Kasper, 2021 [4]	81	M	Tripped	B	Yes	Yes	Yes	ORIF	No
14	Kasper, 2021 [4]	39	M	Fall from bicycle	C	No	Yes	No	EF + ORIF	No
15	Kasper, 2021 [4]	87	F	Tripped at home	C	No	Yes	No	EF + ORIF	No

MOI – mechanism of injury; MMF – medial malleolus fracture; PMF – posterior malleolus fracture, TCF – Tillaux-Chaput fracture, M – male, F – female; ORIF – open reduction internal fixation, SS – syndesmotic screw; EF – external fixator; KW – Kirschner wires.

From this literature review, 9 (60%) were female. Average age was 55 years old (range 87–21). The majority, 11, were classified as Weber C (73%). 2 cases had a delay in diagnosis of the proximal fibular fracture, one case at 3 days post operatively, the other at one week. In both cases of delayed diagnosis, persistent pain prompted treating doctors to further investigate with radiographs.

Prior to the case series of Kasper et al., only 4 previous cases of the ‘Double Maisonneuve’ were described in the literature. Although no exact mechanism has yet been described to explain this fracture pattern, it is thought to be due to an external rotation mechanism owing to the high rates of Weber C level fractures of the distal fibular fracture and the presence of the posterior malleolus fracture. The case described in this paper involved fracture of the posterior malleolus, however, the distal fibular fracture was a B type. From the 15 previous cases, only 4 (26%) of these described B type distal fibular fractures.

This case also highlights the importance of pre-operative CT for ankle malleoli fractures. This has been previously highlighted in the literature by several papers which showed operative plan may altered in many cases based on review of both plain film radiographs and CT versus radiograph alone [14], [15], [16]. This is particularly important for cases involving the posterior malleolus and the Tillaux-Chaput fracture, both present in this case report. Surgical approach or patient positioning, which is extremely difficult to change intraoperatively, has also been shown to change dependant on preoperative CT [17]. The identification of the Tillaux-Chaput fracture in this case was diagnosed only after CT, and may have otherwise been overlooked during surgical planning. Misdiagnosis of such injuries, similar to any intra-articular fracture, can lead to ankle pain, instability and post traumatic arthritis [18].

## Conclusion

The ‘Double Maisonneuve’ fracture pattern, involving fracture of both proximal and distal fibula, is an extremely uncommon injury. Thorough examination and full radiological evaluation are essential when treating an ankle injury. CT is often necessary to fully investigate the ankle preoperatively and to fully understand injury sustained, which will ultimately dictate surgical planning and patient positioning.

## Abbreviations

RA: Rheumatoid arthritis
THA: Total hip arthroplasty
AP: Anteroposterior
CT: Computed tomography
NWB: Non-weight bearing
ORIF: Open reduction internal fixation
DVT: Deep vein thrombosis
FWB: Full weight bearing

## CRediT authorship contribution statement

**Stephen Christopher Murphy:** Writing – original draft, Methodology, Formal analysis, Data curation, Conceptualization. **Gareth Murray:** Data curation. **Adeel Memon:** Writing – review & editing. **Anas El-Sibaei:** Writing – review & editing.

## Consent to publish

Consent was obtained from the patient to be involve in this case report.

## Ethical statement

No formal ethical approval was sought for this paper.

## Funding

No funding was provided for this paper.

## Declaration of competing interest

The authors declare that they have no competing interests.

## Data Availability

All data generated or analysed during this study are included in this published article.
